# Contrasting boundary-layer energy budgets during daytime, nighttime, and compound heatwaves in eastern China

**DOI:** 10.1016/j.isci.2026.114892

**Published:** 2026-02-04

**Authors:** Zexia Duan, Sihui Fan, Yichi Zhang, Tianbo Ji

**Affiliations:** 1 School of Electrical Engineering and Automation, Nantong University, Nantong 226019, China; 2 China Meteorological Administration Xiong’an Atmospheric Boundary Layer Key Laboratory, Hebei 071700, China; 3 China Meteorological Administration Aerosol-Cloud and Precipitation Key Laboratory, Nanjing University of Information, Science and Technology, Nanjing 210044, China; 4 Ningbo Key Laboratory of Smart Meteorology, Ningbo Meteorological Observatory, Ningbo 315000, China; 5 Hangzhou Qian Tang District Meteorological Bureau, Hangzhou 311225, China; 6 School of Transportation and Civil Engineering, Nantong University, Nantong 226019, Jiangsu, China

**Keywords:** Earth sciences, Environmental science, heat transfer

## Abstract

Heatwaves over the Yangtze River Delta (YRD) have intensified in frequency and diversity amid rapid urbanization and climate change, yet their underlying boundary-layer processes remain insufficiently understood. This study examines boundary-layer energy budgets of three heatwave types across the YRD using observations from 186 meteorological stations and reanalysis data covering 2002–2022. Daytime-only heatwaves exhibited reduced cloudiness (−37%), enhancing shortwave radiation (+201 W/m^2^), while efficient nocturnal cooling limited nighttime warming. Nighttime-only events were characterized by greater cloudiness (+6%) and humidity, increasing longwave radiation and suppressing cooling, with rice paddies buffering daytime temperature extremes more effectively than forests. Compound heatwaves combined both mechanisms—enhanced daytime solar heating and inhibited nocturnal cooling—producing the most intense temperature anomalies (>10 °C) concentrated in coastal-urban areas. Enhanced thermal turbulence deepened the boundary layer during daytime and compound events, whereas mechanical turbulence weakened under subsidence. These findings clarify heatwave boundary-layer processes and support region-specific climate adaptation strategies.

## Highlights

• Daytime heatwaves driven by reduced clouds and enhanced shortwave radiation
• Nighttime heatwaves caused by moisture-cloud feedback enhancing longwave radiation
• Compound heatwaves combine solar heating and inhibited cooling to maximize intensity
• Land surface heterogeneity modulates heatwave energy budgets

## Graphical abstract

**Figure:**
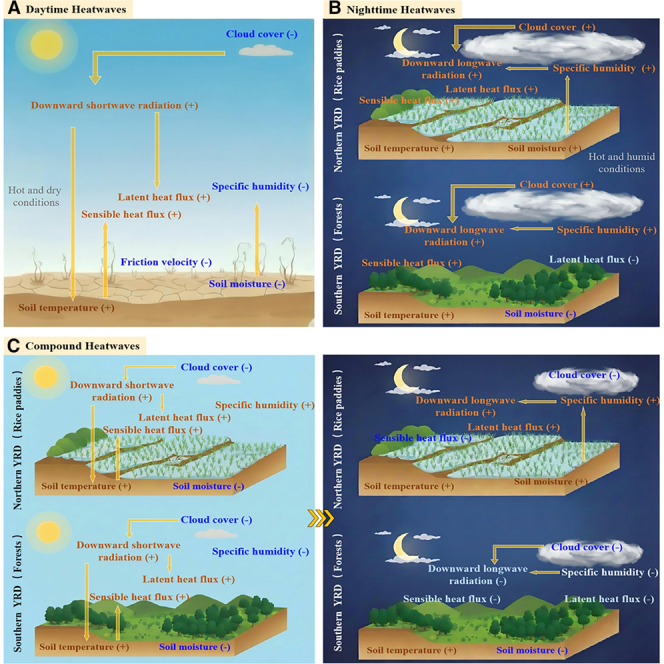

## Introduction

**Figure 1:**
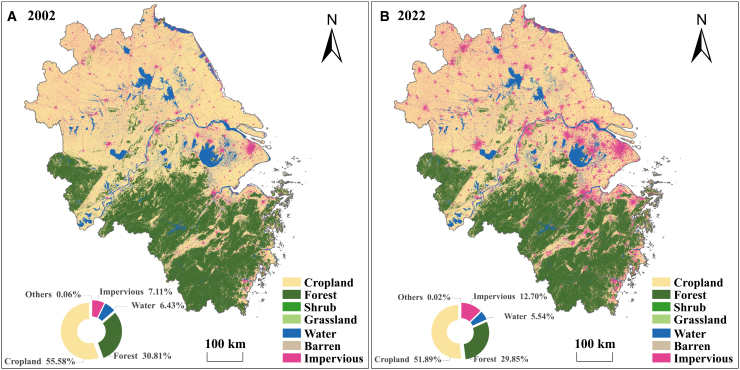
Spatial distribution of land use/land cover in the Yangtze River Delta (A) Distribution pattern in 2002. (B) Distribution pattern in 2022. The donut charts in the inset illustrate the percentage area of each land cover type. Scale bars, 100 km.

Heatwaves represent one of the most deadly weather extremes, causing thousands of fatalities annually and imposing substantial burdens on public health, energy systems, and economic productivity.^1^^,^^2^ Under ongoing climate change, heatwave frequency, intensity, and duration are projected to increase globally, with particularly pronounced impacts expected across densely populated regions of East Asia.^3^ The Yangtze River Delta (YRD)—including Shanghai, Jiangsu, Anhui, and Zhejiang—exemplifies this vulnerability. Geographically, the region exhibits a distinct and heterogeneous spatial pattern of land cover (Figure 1). The northern part is dominated by cropland, specifically rice-wheat rotation systems (e.g., flooded rice paddies in summer and wheat fields in winter), accounting for over 50% of the total area. In contrast, the southern part is characterized by dense forests and hilly landscapes (e.g., in Zhejiang), covering approximately 30% of the region. Over the past two decades, the YRD has undergone rapid urbanization. Comparing land cover data from 2002 to 2022 (Figures 1A and 1B), the coverage of impervious surfaces expanded significantly from 7.11% to 12.70%, primarily encroaching upon cropland, which decreased from 55.58% to 51.89%. Despite these rapid changes, the fundamental spatial heterogeneity—cropland in the north and forest in the south—remains intact. This complex land cover, combined with intense human activity, significantly influences surface energy partitioning and land-atmosphere moisture feedback,^4^ amplifying the region’s exposure and sensitivity to heat-related impacts.^5^

Recent decades have witnessed intensifying heat extremes across the YRD, with record-breaking temperatures observed during the summers of 2013, 2017, and 2022.^6^^,^^7^ Previous research on heatwaves in the YRD has predominantly focused on attribution analyses, examining the roles of large-scale atmospheric circulation patterns, anthropogenic warming, and urbanization effects.^8^^,^^9^^,^^10^^,^^11^ Additionally, considerable attention has been devoted to assessing heatwave impacts on ecosystems, including suppressed vegetation growth and disrupted carbon cycling.^12^^,^^13^^,^^14^^,^^15^ Other studies have advanced forecasting capabilities, developed risk assessment frameworks, and quantified health burdens, revealing disproportionate impacts on elderly and socioeconomically disadvantaged populations.^16^^,^^17^

Although existing literature has elucidated the characteristics and impacts of heatwaves, focus has largely remained on daytime events driven by extreme maximum temperatures.^18^ These events are mechanistically linked to persistent anomalous anticyclones,^19^ where quasi-stationary blocking highs and Rossby wave trains induce subsidence, warm advection, and radiative heating via cloud suppression.^20^^,^^21^^,^^22^ Conversely, nighttime heatwaves, characterized by elevated minimum temperatures, present unique physiological hazards by preventing nocturnal recovery.^23^^,^^24^ These events are thermodynamically distinct, predominantly driven by atmospheric moisture anomalies that enhance downward longwave radiation.^25^ Studies across the western United States^26^ and East Asia^27^^,^^28^ identify precipitable water and moisture advection as critical drivers of nighttime heat intensity. An emerging and critical concern is the compound heatwave, which combines extreme daytime and nighttime temperatures, thereby creating continuous thermal stress with negligible relief.^29^ Compared to single-type events, the genesis of compound heatwaves has received limited attention. Observations in China and North America suggest these events arise from persistent anticyclones with quasi-barotropic or stable baroclinic structures that promote low-level moisture convergence.^30^^,^^31^ This moisture accumulation suppresses nocturnal radiative cooling, maintaining high temperatures throughout the diurnal cycle. Since the synoptic forcing for daytime, nighttime, and compound heatwaves exhibits significant regional heterogeneity, it is essential to rigorously differentiate these types and disentangle their underlying physical mechanisms.

Understanding the physical mechanisms underlying different heatwave types requires examining the atmospheric boundary layer (ABL) processes that govern near-surface temperature variations. The ABL, defined as the lowest part of the atmosphere directly influenced by interactions with the Earth’s surface,^32^ plays a critical role in regulating the near-surface thermal environment. The heat budget of the ABL has significant implications for both ecosystem functioning and human health.^33^ Previous studies have demonstrated that the thermal structure of the ABL is highly sensitive to heatwave events and exhibits significant diurnal variations.^34^^,^^35^ The ABL heat budget processes are complex and diverse, encompassing turbulent diffusion, radiative transfer, large-scale advection, microphysical processes, and cumulus convection.^36^^,^^37^ Furthermore, the efficiency and partitioning of energy through these pathways are strongly modulated by the underlying land surface characteristics. However, how this surface heterogeneity—such as the pronounced contrast between the agricultural north and forested south of the YRD—alters the ABL energy budget during different types of heatwaves remains poorly quantified.

Thus, this study integrates Automatic Weather Station observations with high-temporal-resolution (2002–2022) reanalysis data for the YRD to (1) characterize and compare the spatiotemporal patterns of daytime-only, nighttime-only, and compound heatwaves, revealing their divergent occurrence characteristics and evolution trends; (2) quantify the differential contributions of radiation-moisture coupling, surface energy partitioning, and boundary layer dynamics to each heatwave type; and (3) elucidate how land surface heterogeneity, particularly the contrast between northern agricultural and southern forested regions, modulates these energy budget processes. The novelty of this work lies in its systematic comparative analysis of three different heatwave types through integrated boundary layer energetics across the heterogeneous YRD landscape. Our key contributions include (1) identifying specific radiation-moisture feedback mechanisms that differentiate daytime (shortwave driven with dry conditions), nighttime (longwave driven with moist conditions), and compound (hybrid with spatial heterogeneity) heatwaves; (2) revealing vegetation-dependent surface energy responses, particularly the contrasting behaviors of rice paddies versus forests in modulating sensible heat flux (SHF) and latent heat flux (LHF); and (3) demonstrating the opposing roles of thermal versus mechanical turbulence in heatwave amplification. These findings advance fundamental understanding of heatwave physics and provide critical insights for improving heatwave prediction and developing targeted adaptation strategies for the vulnerable YRD region.

## Results

### Spatiotemporal distribution of heatwaves

**Figure 2:**
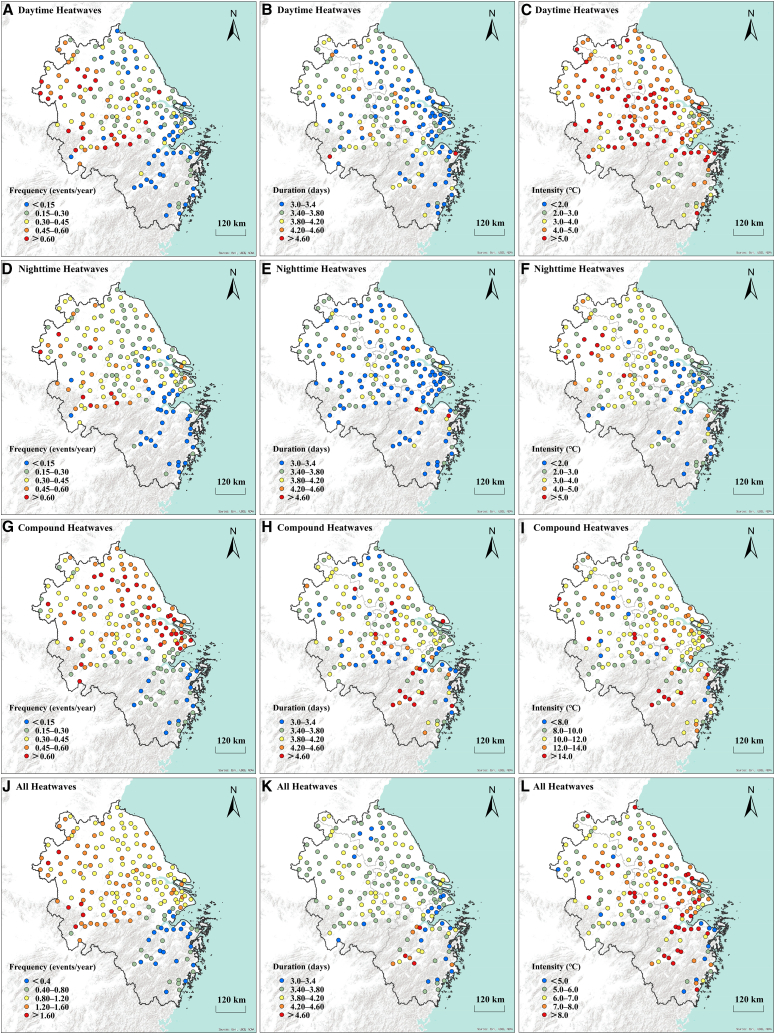
Spatial patterns of different heatwave types in the Yangtze River Delta (2002–2022) (A–C) Daytime-only heatwaves. (D–F) Nighttime-only heatwaves. (G–I) Compound heatwaves. (J–L) All heatwaves. In all rows, the columns from left to right display mean frequency, duration, and intensity, respectively. Scale bars, 120 km.

Figure 2 presents the station-level spatial distribution of the average frequency, duration, and intensity of daytime-only, nighttime-only, and compound heatwaves in the YRD from 2002 to 2022. In Figure 2A, the frequency of daytime-only heatwaves exceeds 0.6 events/year in the northwestern YRD, while Figure 2B shows prolonged duration (>3.4 days) in similar regions. The intensity of daytime-only heatwaves peaks (>5°C) in the central and northern YRD (Figure 2C). Nighttime-only heatwaves exhibit marked spatial patterns. The frequency is lowest in Zhejiang province, with an annual average of <0.15 events/year, while higher frequencies are observed in the central and northern YRD (Figure 2D). The duration is relatively uniform across the region, ranging between 3 and 4.2 days (Figure 2E). The intensity is highest in the central and northern YRD, exceeding 2°C (Figure 2F). Compound heatwaves (Figures 2G–2I) display the highest frequencies and duration in coastal and urbanized regions, with extreme intensities (>10°C) concentrated in the east and southeast (Figure 2I). The aggregated analysis (Figures 2J–2l) reveals widespread event occurrence (Figure 2J), prolonged duration in the northern and central areas (Figure 2K), and severe intensities (>8°C) along the eastern coastline (Figure 2L). These results highlight the spatial heterogeneity of heatwaves, emphasizing the heightened vulnerability of coastal and urban areas to extreme events.

**Figure 3:**
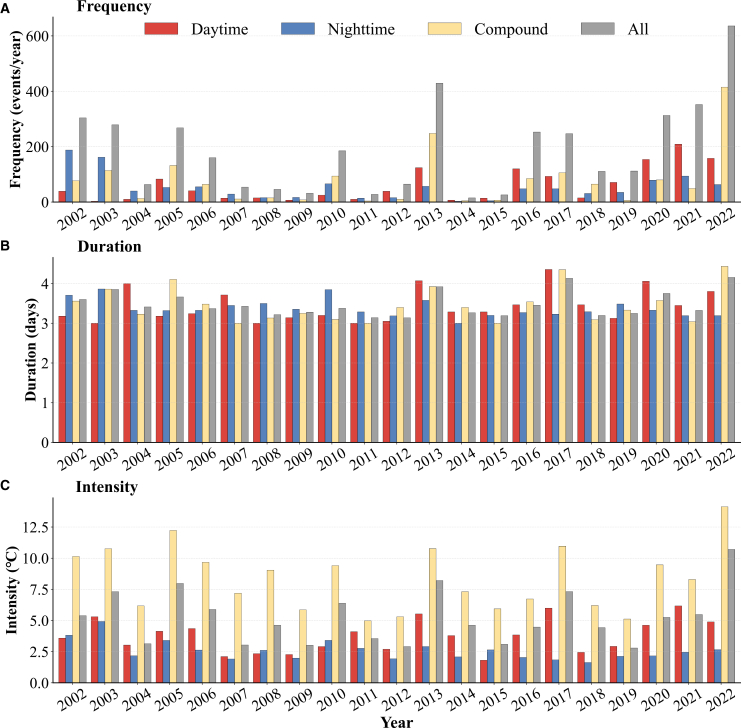
Interannual variations in heatwave characteristics across the Yangtze River Delta (2002–2022) (A) Frequency of heatwaves. (B) Duration of heatwaves. (C) Intensity of heatwaves. Colors indicate daytime-only (red), nighttime-only (blue), compound (yellow), and all (gray) heatwaves. Data represent the regional mean values for each category.

Figure 3 presents annual regional means of heatwave (1) frequency, (2) duration, and (3) intensity in the YRD during 2002–2022 for daytime-only, nighttime-only, compound, and all events. Daytime-only heatwaves show a clear upward trend in frequency, with a maximum in 2021, while duration is relatively stable around 3.0–4.5 days. The intensity of daytime-only heatwaves has increased gradually, reaching approximately 6°C in recent years. Nighttime-only heatwaves display a generally decreasing or fluctuating frequency with intermittent peaks (e.g., 2002–2003), nearly constant duration of 3.0–3.5 days, and intensities that occasionally approach 4°C without an obvious long-term trend. Compound heatwaves exhibit a pronounced rise in frequency, particularly in 2022; durations are comparable to other categories (3.0–4.5 days), whereas intensity varies widely and can reach up to ∼15°C. When all events are combined, frequency shows a distinct increase culminating in 2022, duration remains stable, and intensity rises steadily to approximately 5°C–10°C in recent years.

### Radiation-moisture changes during heatwaves

**Figure 4:**
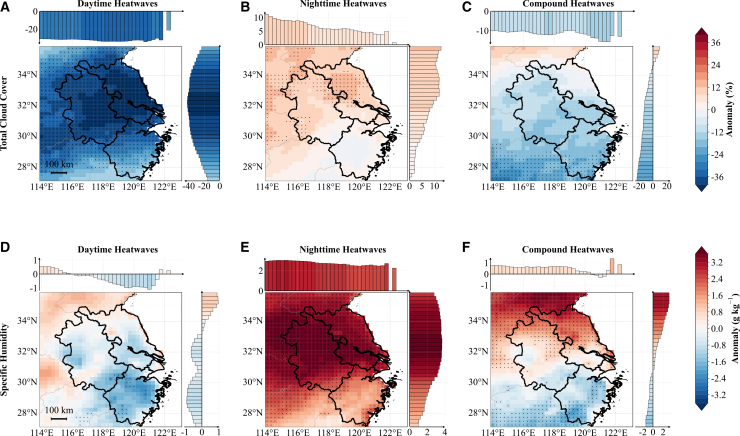
Spatial composite anomalies during daytime hours (11:00–16:00 local time) for three heatwave types over the YRD, China (A–C) Total cloud cover anomalies. (D–F) Near-surface specific humidity anomalies. Columns from left to right correspond to daytime-only, nighttime-only, and compound heatwaves. Black stippling on maps marks grid cells where anomalies are statistically significant at the 95% confidence level according to a Student’s *t* test. Bar plots above and to the right of each map show zonal (longitude-dependent) and meridional (latitude-dependent) variations of the anomalies, respectively. The maps and accompanying bar plots share the same color bar. Data are represented as the mean anomalies. Scale bars, 100 km.

**Figure 5:**
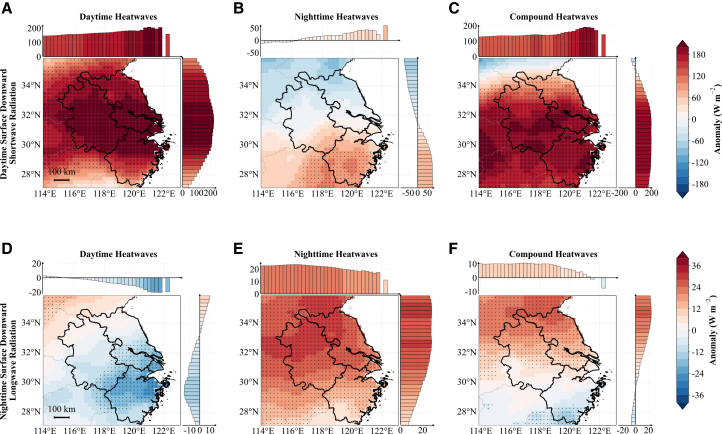
Spatial composite anomalies of surface radiation for three heatwave types over the YRD, China (A–C) Daytime (11:00–16:00 local time) surface downward shortwave radiation anomalies. (D–F) Nighttime (23:00–04:00 local time) surface downward longwave radiation anomalies. Columns from left to right correspond to daytime-only, nighttime-only, and compound heatwaves, respectively. Stippling, bar plots, scale bars, and color bar are as in Figure 4. Data represent the composite mean values.

**Figure 6:**
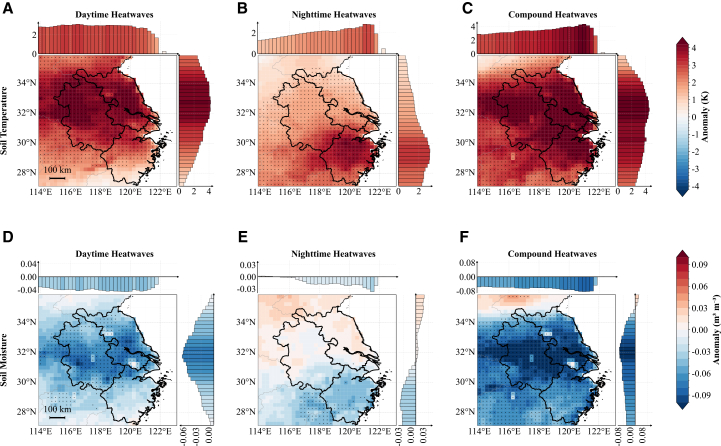
Daytime (11:00–16:00 local time) soil temperature and soil moisture anomalies (A–C) Soil temperature anomalies. (D–F) Soil moisture anomalies. In all rows, the columns from left to right correspond to daytime-only, nighttime-only, and compound heatwaves, following the same layout as in Figure 4.

**Figure 7:**
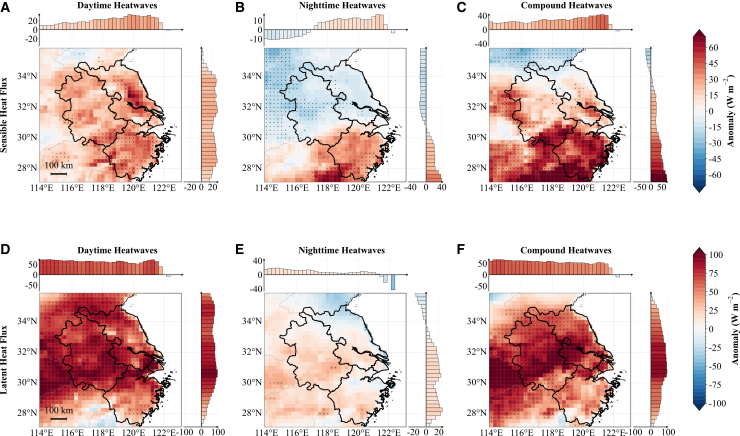
Daytime (11:00–16:00 local time) sensible heat flux and latent heat flux anomalies (A–C) Sensible heat flux anomalies. (D–F) Latent heat flux anomalies. In all rows, the columns from left to right correspond to daytime-only, nighttime-only, and compound heatwaves, following the same layout as in Figure 4.

**Figure 8:**
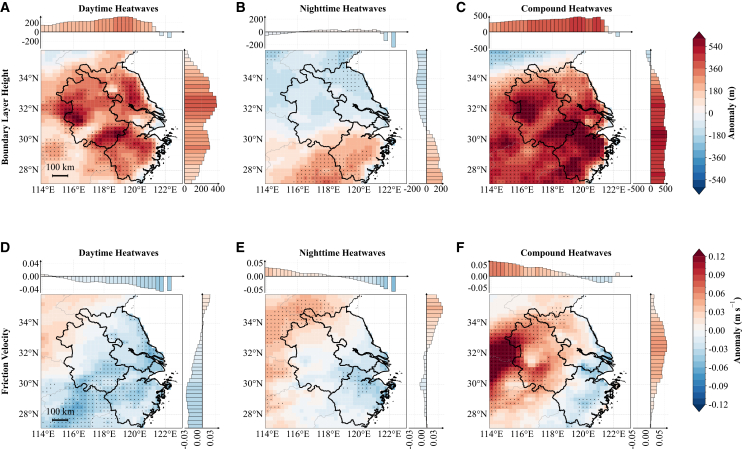
Daytime (11:00–16:00 local time) boundary layer height and friction velocity anomalies (A–C) Boundary layer height anomalies. (D–F) Friction velocity anomalies. In all rows, the columns from left to right correspond to daytime-only, nighttime-only, and compound heatwaves, following the same layout as in Figure 4.

Figure 4 shows daytime (11:00–16:00 local time) composite anomalies of total cloud cover and near-surface specific humidity for daytime-only, nighttime-only, and compound heatwaves over the YRD. Nighttime (23:00–04:00 local time) composites with analogous spatial patterns are provided in Figure S1. For each map, bar plots above and to the right display zonal (longitude-dependent, latitudinal mean) and meridional (latitude-dependent, longitudinal mean) anomalies. The same visualization is used for Figures 4, 5, 6, 7, 8, and S1–S4. Figure 5 illustrates the associated anomalies in daytime downward shortwave and nighttime downward longwave radiation.

Daytime-only heatwaves are characterized by a marked reduction in cloud cover over the YRD during daytime hours (11:00–16:00 local time; −37% averaged over the YRD; Figure 4A), which allows more solar shortwave radiation to reach the surface (∼201 W m^−2^ increase; Figure 5A), thus directly increasing near-surface temperatures. Concurrently, daytime near-surface specific humidity decreases substantially (Figure 4D), indicating a drier boundary layer. This reduction in atmospheric moisture further enhances solar radiation heating of the air near the surface, as the dry and hot conditions with less cloud cover and more solar radiation create a favorable environment for daytime heatwave occurrence and maintenance, consistent with previous findings for southern China.^28^ Notably, cloud cover also decreases during nighttime hours (23:00–04:00 local time; Figure S1A), which weakens downward atmospheric longwave radiation and enhances nocturnal surface longwave cooling. Although nighttime specific humidity increases in the northern YRD, it still shows a decreasing trend in the forest-dominated southern region (Figure S1D). Crucially, the magnitude of cloud cover reduction during daytime-only events is substantially larger (−37%) compared to compound events (−12%). This extreme clear-sky condition creates a dominant radiative cooling effect that overpowers the local greenhouse effect of increased atmospheric moisture over the northern YRD. Combined with the enhanced radiative cooling from reduced cloudiness, this effectively dissipates the accumulated daytime heat, limiting the carryover of daytime warmth into the night and thus yielding “pure” daytime events rather than compound heatwaves.

Nighttime-only heatwaves exhibit distinct radiative characteristics. During the preceding daytime hours (11:00–16:00 local time), cloud cover increases moderately (+6% averaged over the YRD; Figure 4B) and specific humidity is elevated across the YRD (Figure 4E). This enhanced cloudiness significantly weakens downward solar radiation at the surface—the daytime enhancement in shortwave radiation is only +17 W m^−2^ (Figure 5B), much weaker than during daytime heatwaves. This indicates that nighttime heatwaves are triggered by longwave radiation rather than shortwave radiation. The increased cloud cover and atmospheric moisture enhance downward longwave radiation at the surface (Figure 5E), warming the near-surface air layer. Additionally, the elevated water vapor intensifies the greenhouse effect, trapping upward longwave radiation. During nighttime hours (Figure S1B), these cloud-moisture anomalies are particularly effective at suppressing radiative cooling, maintaining anomalously warm temperatures. This mechanism is consistent with previous studies documenting similar processes in other regions including southern China, the contiguous United States, and the Korean Peninsula.^3^^,^^27^^,^^38^

Compound heatwaves exhibit hybrid characteristics with distinct spatial heterogeneity across the YRD. Area-mean cloud cover decreases by 12% (Figure 4C), substantially increasing daytime downward shortwave radiation (∼169 W m^−2^; Figure 5C), similar to daytime-only heatwaves. However, specific humidity shows a pronounced north-south dipole pattern throughout the diurnal cycle, with positive anomalies in northern YRD and negative anomalies in southern YRD (Figure 4F). This meridional contrast translates into corresponding nighttime downward longwave radiation patterns (Figure 5F), with enhanced downward longwave radiation in the north and reduced radiation in the south. This spatial heterogeneity likely reflects differences in land surface characteristics—the northern YRD is dominated by agricultural lands with extensive irrigation systems that enhance evapotranspiration and maintain higher atmospheric moisture levels, whereas the southern YRD features more forested areas with different moisture flux patterns. In the north, enhanced atmospheric moisture increases atmospheric emissivity and suppresses nocturnal radiative cooling, maintaining elevated nighttime temperatures. Conversely, the relatively drier conditions in the south allow for greater radiative cooling, although the reduced cloud cover still ensures substantial daytime heating. This dual mechanism—uniform enhancement of daytime solar heating from reduced clouds combined with spatially heterogeneous moisture-modulated radiative processes—creates a complex thermal environment that sustains anomalously high temperatures throughout the diurnal cycle, with more intense compound effects in the northern agricultural regions.

### Response of surface energy exchange to heatwaves

Figures 6 and 7 examine the land surface and turbulent flux responses to three heatwave regimes, complementing the radiation-moisture mechanisms described previously. Surface SHF and LHF are defined positive upward; thus, positive anomalies indicate enhanced upward energy transfer from the surface to the atmosphere.

During daytime hours (11:00–16:00 local time), soil temperature increases across the YRD, with area-mean anomalies of approximately +3.4 K for daytime-only heatwaves, +2.3 K for nighttime-only heatwaves, and up to +4.0 K for compound heatwaves (Figures 6A–6C). The soil moisture response reveals contrasting spatial patterns that depend on the heatwave regime. Daytime-only and compound heatwaves produce widespread soil moisture depletion across most of the YRD region (Figures 6D and 6F). In contrast, nighttime-only heatwaves generate a distinctive dipole pattern in soil moisture anomalies (Figure 6E): the northern YRD, dominated by rice paddies, experiences modest moisture increases, whereas the southern forested regions exhibit moisture deficits comparable to those observed during other heatwave types. During nighttime hours (23:00–04:00 local time), all heatwave types display spatial patterns similar to their daytime counterparts but with reduced amplitudes (Figure 6; see also Figure S2).

These contrasting soil temperature and moisture patterns drive differential surface energy partitioning across distinct heatwave regimes. Figure 7 illustrates the spatial distribution of SHF and LHF anomalies across the YRD during daytime hours (11:00–16:00 local time). During daytime-only and compound heatwaves (Figures 7A and 7C), upward SHF intensifies across the majority of the YRD, exhibiting positive area-mean anomalies of 28 W m^−2^ and 29 W m^−2^, respectively. This intensification is primarily driven by enhanced temperature gradients between the heated land surface and the overlying atmosphere. Conversely, nighttime-only heatwaves reveal a pronounced north-south dichotomy (Figure 7B): northern rice paddy regions exhibit suppressed upward SHF (negative anomalies), whereas southern forested areas maintain enhanced upward sensible heat transfer. LHF consistently shows enhanced upward transfer (positive anomalies) across all heatwave types (Figures 7D–7F), with the most pronounced intensification observed during daytime-only and compound events.

In contrast to daytime dynamics, the magnitude of nocturnal turbulent fluxes (23:00–04:00 local time) is generally weaker (Figure S3). Regarding nocturnal SHF, both daytime-only and compound heatwaves exhibit widespread negative anomalies, whereas nighttime-only heatwaves show weaker, positive, and spatially heterogeneous responses. For nocturnal LHF, compound heatwaves display significant positive anomalies in the central-northern regions; this enhanced nocturnal evaporation exacerbates heat stress through humidification and the water vapor greenhouse effect. The other two heatwave types show either negative anomalies in the south or weak positive LHF responses in the north, with nighttime-only heatwaves notably suppressed in coastal areas.

This differential response reflects the distinct thermal and hydrological properties of the underlying land cover types. Rice paddies, characterized by high water availability and substantial thermal inertia, effectively buffer temperature extremes and maintain elevated soil moisture levels during the daytime of nighttime-only heatwaves, thereby suppressing sensible heat exchange with the atmosphere. In contrast, forested areas, which typically exhibit lower soil moisture availability, experience persistent moisture stress that sustains strong sensible heat transfer during the daytime, even under nighttime-only heatwave conditions. These land cover-mediated feedbacks underscore the critical role of surface heterogeneity in modulating regional responses to different heatwave regimes and highlight the importance of considering land surface characteristics when assessing heatwave impacts and mechanisms.

### Boundary-layer height and friction velocity variation

To further investigate the dynamical features of heatwaves over the YRD, we analyze boundary-layer height (BLH) and friction velocity (*u∗*) anomalies during daytime (11:00–16:00 local time) and nighttime (23:00–04:00 local time) periods (Figures 8 and S4). BLH reflects the combined effects of thermal and mechanical turbulence. During heatwaves, however, daytime BLH anomalies are primarily driven by enhanced thermal convection. *u∗* directly measures mechanically driven surface momentum exchange, responding primarily to near-surface wind speed and shear conditions.

During the daytime period (11:00–16:00 local time) of daytime-only heatwaves, the YRD exhibits strong positive BLH anomalies (Figure 8A), indicating enhanced thermal convection and boundary-layer deepening. This deep mixing layer entrains warm air from aloft, amplifying surface warming. Concurrently, *u∗* shows weak negative anomalies across most of the region (Figure 8D), suggesting that large-scale subsidence reduces near-surface winds and weakens mechanical turbulence despite strengthened thermal convection. Nighttime-only heatwaves evaluated during daytime show a distinct north-south dipole in BLH, with negative anomalies in the north and positive in the south (Figure 8B). This spatial contrast is mechanistically linked to the land cover heterogeneity analyzed earlier. In the northern YRD, the dominant rice paddies suppress SHF (Figure 7B) due to high evapotranspiration, thereby limiting thermal buoyancy and inhibiting boundary layer growth. Conversely, the southern forested regions maintain positive SHF anomalies, driving stronger thermal turbulence that deepens the boundary layer. *u∗* anomalies during nighttime-only heatwaves (Figure 8E) are near neutral in the northern YRD and negative in the south, indicating localized weakening of surface turbulence. Compound heatwaves produce the strongest BLH enhancement (Figure 8C), reflecting intense convective mixing throughout the YRD. The corresponding *u∗* exhibits a clear spatial gradient (Figure 8F)—positive in the north but weakly negative in the south. Although thermal turbulence is universally enhanced during compound events, this *u∗* gradient likely reflects the aerodynamic roughness differences. The open, flat agricultural terrain in the north facilitates momentum transfer under the intensified thermal instability, whereas the complex topography and forest canopy in the south may modulate near-surface wind shear differently under subsidence conditions.

At night (23:00–04:00 local time00), BLH anomalies generally reverse from daytime patterns. Daytime-only heatwaves show negative anomalies across most areas, particularly along the southern coast, indicating nocturnal boundary layer suppression under stable stratification (Figure S4A). Compound heatwaves exhibit similar negative anomalies, strongest in the south, while northern positive anomalies are scattered, weak, and statistically insignificant (Figure S4C). Nighttime-only heatwaves display slight positive anomalies over the northern/northwestern YRD, although these lack magnitude, spatial coherence, and statistical robustness; southern areas show consistent negative anomalies (Figure S4B). Nighttime *u∗* anomalies are smaller than daytime values but retain pronounced spatial contrasts. Daytime-only heatwaves produce slightly negative *u∗* anomalies across southern and coastal YRD, while weak positive pockets are observed in the north (Figure S4D). Nighttime-only events show predominantly positive northern/northwestern anomalies that mostly fail significance tests (sparse stippling), indicating low confidence, while southern areas exhibit near-zero to slightly negative values (Figure S4E). Compound heatwaves present a similar but weaker north-south dipole with largely non-significant northern anomalies (Figure S4F). Overall, heatwaves produce asymmetric coastal-inland boundary-layer responses: statistically robust BLH suppression and weakened surface mixing occur consistently in coastal/southern areas (strongest during compound events), whereas inland/northern areas show weak, spatially inconsistent enhancement tendencies that generally lack significance. This suggests that heatwave-induced boundary-layer modifications are more reliable in coastal regions.

BLH and *u∗* respond differently to heatwaves because they reflect distinct turbulence processes. During the daytime hours of daytime-only and compound heatwaves, thermal turbulence tends to deepen the boundary layer and enhance entrainment, which can intensify surface warming. Meanwhile, mechanical turbulence often weakens as large-scale subsidence reduces near-surface wind speeds. At night, boundary-layer suppression is generally more common, except during nighttime-type heatwaves, when accumulated heat may maintain partial mixing in some areas. Although nighttime *u∗* anomalies are typically weak, localized positive signals in the northern YRD suggest a possible persistent mechanical contribution. Taken together, these contrasting responses indicate that thermal turbulence likely plays the primary role in amplifying heatwaves, whereas mechanical turbulence mainly modulates spatial variability across the YRD, with notable regional and statistical uncertainties.

## Discussion

### Mechanisms of different heatwave types

**Figure 9:**
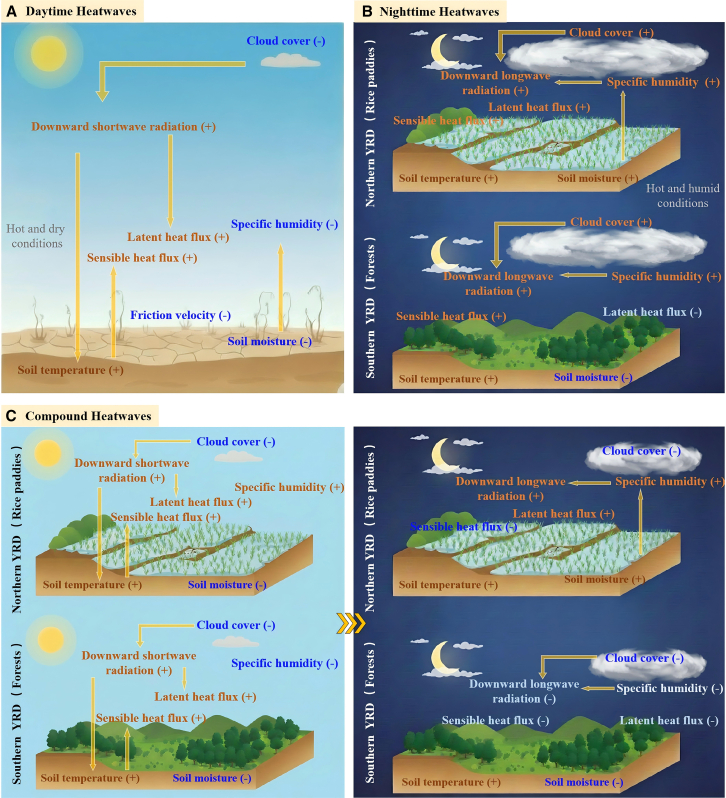
Conceptual schematic diagram of physical processes during different heatwave types (A) Daytime-only heatwaves under hot-dry conditions. (B) Nighttime-only heatwaves under hot-humid conditions. (C) Compound heatwaves. (B and C) illustrate the differences between the northern YRD (rice paddy dominated) and the southern YRD (forest dominated). The symbols (+) and (−) denote increases (positive anomalies) and decreases (negative anomalies).

Our analysis reveals distinct physical mechanisms underlying the three heatwave types in the YRD region, as synthesized in Figure 9. Daytime-only heatwaves are primarily driven by enhanced shortwave radiation under clear-sky conditions (Figure 9A). The substantial reduction in cloud cover (−37%) allows increased solar radiation to reach the surface, while decreased atmospheric moisture and soil water content amplify SHF. The increased BLH facilitates entrainment of warm air from aloft, further intensifying surface warming. However, the reduced cloud cover at night enhances longwave cooling, preventing the persistence of high temperatures into nighttime hours. This distinguishes daytime-only events from compound heatwaves: despite the presence of agricultural moisture in the northern YRD, the “atmospheric window” remains open due to the severe lack of cloud cover, allowing outgoing longwave radiation to escape efficiently and preventing the nocturnal heat trapping observed in compound events.

In contrast, nighttime-only heatwaves are characterized by a fundamentally different mechanism dominated by longwave radiation trapping (Figure 9B). The increased cloud cover and atmospheric humidity during both day and night enhance downward longwave radiation, creating a greenhouse effect that suppresses nocturnal cooling. The moderate daytime cloud cover limits solar heating, explaining why these events do not manifest as daytime extremes. Furthermore, land-atmosphere feedbacks over the agricultural northern YRD play a critical buffering role. The extensive rice paddies, characterized by saturated soil conditions, partition available energy primarily into LHF rather than SHF. This strong evaporative cooling prevents daytime temperatures from rising sharply. However, the associated accumulation of near-surface moisture reinforces the local greenhouse effect, effectively preventing heat escape during the night. Consequently, the daytime boundary layer in these agricultural regions exhibits negative height anomalies due to suppressed thermal buoyancy, which limits the vertical dilution of moisture and heat, thereby sustaining the anomalously high nocturnal temperatures.

Compound heatwaves exhibit hybrid characteristics that sustain extreme temperatures throughout the diurnal cycle. These events combine the clear-sky solar heating of daytime heatwaves with selective moisture-enhanced longwave trapping, particularly in the northern agricultural regions. The spatial heterogeneity in moisture patterns—wet in the north, dry in the south—creates differential nocturnal cooling rates that modulate the intensity and spatial extent of compound events.

### Vegetation-mediated responses to heatwaves

Our findings reveal distinct ecosystem-specific responses to heatwaves in the YRD, contrasting significantly with observations from European studies (e.g., Tian et al., 2024).^39^ In the YRD, a pronounced divergence exists between northern rice paddies and southern forests. During the daytime phase of nighttime-only heatwaves, northern rice paddies exhibit suppressed or negative SHF anomalies. This response effectively buffers daytime warming, preventing maximum temperatures from breaching extreme thresholds. Conversely, southern forests maintain positive SHF anomalies. However, this pattern reverses at night in the north, where positive SHF anomalies contribute to elevated minimum temperatures.

The primary driver of this divergence—and the key distinction from European observations—is agricultural water management. The YRD’s croplands in summer are predominantly flooded rice paddies, where standing water and saturated soils provide substantial evaporative cooling capacity (Bowen ratio <1).^40^ This persistent water availability allows rice paddies to suppress SHF increases even during extreme heat, creating a “cool island” effect. In contrast, the European croplands reported by Tian et al. (2024)^39^ are primarily rainfed systems (e.g., wheat, maize). These systems experience rapid soil moisture depletion during heatwaves, constraining evapotranspiration and forcing energy dissipation through increased SHF. Thus, while European crops amplify heating through moisture stress, YRD rice paddies mitigate daytime extremes through irrigation-sustained LHF.

Forest responses further underscore regional hydroclimatic distinctions. While forests in both regions generally exhibit increased SHF during heatwaves, the underlying mechanisms differ. The evergreen broadleaf and mixed forests of the southern YRD maintain high SHF due to deep root systems and year-round physiological activity, partitioning substantial energy into sensible heat even during humid, nighttime-only heatwaves. European forests, often deciduous or coniferous, display more variable responses governed by phenology and seasonal water strategies.

These land surface anomalies significantly influence planetary boundary layer (PBL) dynamics. The suppressed SHF over northern rice paddies limits vertical PBL development, whereas the sustained heating over southern forests promotes deeper mixing layers. Consequently, the agricultural north and forested south experience distinct atmospheric ventilation conditions. These findings highlight the critical role of land cover and management practices in shaping regional climate feedbacks. While the cooling effect of rice paddies suggests irrigation as a potential heat mitigation strategy, this benefit must be balanced against water resource constraints and the risk of enhanced nighttime warming due to increased humidity. Future research should explicitly account for crop-specific management and phenology to accurately evaluate land-atmosphere feedback under warming climates.

### Synergistic effects of urbanization and heatwaves

Our results indicate that coastal-urban areas, such as Shanghai, exhibit significantly higher heatwave intensity and duration compared to their rural counterparts (Figure 2), particularly during compound heatwave events. This spatial disparity highlights the critical role of the urban heat island effect in amplifying regional heatwaves.^41^ Unlike the agricultural landscapes (e.g., rice paddies) and forests discussed previously, urban areas are characterized by a dominance of impervious surfaces—such as asphalt and concrete—which possess distinct thermal properties that fundamentally alter the surface energy budget.^42^

First, impervious surfaces typically have lower albedo and higher thermal admittance compared to natural vegetation. During the daytime, these artificial materials absorb and store a substantial amount of incoming solar radiation as ground heat flux.^10^ Crucially, due to the scarcity of surface moisture and vegetation, urban surfaces exhibit a significantly higher Bowen ratio, channeling the majority of available net radiation into SHF rather than LHF.^43^ This mechanism directly drives up near-surface air temperatures and contrasts sharply with the evaporative cooling observed in the northern rice paddies.

Second, the heat stored within the urban fabric during the day is released gradually at night, effectively preventing the nocturnal cooling that typically occurs in rural environments.^44^ This retention of heat is exacerbated by the “urban canyon effect” created by high-rise buildings, which reduces the sky view factor and traps outgoing longwave radiation.^25^ This process is particularly relevant to our findings on compound heatwaves, where suppressed nocturnal cooling is identified as a key driver of heat stress. Furthermore, the release of anthropogenic heat from air-conditioning systems and vehicular traffic further intensifies this warming.^45^

Finally, the boundary layer dynamics over urban areas differ markedly from those over rural lands. The intense sensible heating can lead to a deeper, yet significantly warmer, urban boundary layer during the day.^10^ The interaction between this urban heat island effect and the background synoptic heatwave creates a synergistic amplification, further deepening the urban boundary layer and preventing nocturnal recovery. During the night, the persistent release of surface heat maintains a neutral or unstable residual layer, preventing the formation of the stable stratification typically observed over rural forests.^46^ These findings underscore the necessity of integrating urban planning strategies to mitigate heat risks in high-density coastal zones.^47^

### Limitations of the study

This study acknowledges several methodological limitations that warrant consideration. First, the coarse spatial resolution of the ERA5 reanalysis data (0.25° × 0.25°) constrains the capacity to resolve small-scale surface heterogeneity. Despite rapid urbanization in the YRD, where impervious surfaces increased to 12.70% by 2022, urban areas remain spatially fragmented (Figure 1). Consequently, ERA5 grid cells inevitably integrate signals from diverse land covers. To mitigate this “mixed-pixel” effect, the analysis focused on the agricultural and forest ecosystems that dominate over 80% of the region, as they govern the bulk of the regional land-atmosphere energy exchange. While eddy covariance flux towers could provide high-frequency, ecosystem-specific measurements, they were excluded due to the difficulty in upscaling point measurements to the regional level and the lack of continuous records covering the full 21-year study period.

Second, the study period (2002–2022) is relatively short for climatological analysis. While 21 years of data are adequate for identifying the physical mechanisms distinguishing heatwave types, this time frame may not fully capture multi-decadal variability or long-term intensification trends associated with broader climate change signals.

Third, the scope of this work is limited to historical mechanism identification and does not include future climate projections. Specifically, detection-and-attribution studies or CMIP6-based modeling were not incorporated. Future research should extend the mechanistic framework established here to climate model outputs to assess the evolution of compound and single-type heatwaves under various warming scenarios.

## Resource availability

### Lead contact

Requests for further information and resources should be directed to and will be fulfilled by the lead contact, Sihui Fan (sihuif_1017@163.com).

### Materials availability

This study did not generate new unique reagents.

### Data and code availability

• Data: ERA5 reanalysis data from the European Center for Medium-Range Weather Forecasts (ECMWF) are freely available for download through the Copernicus Climate Change Service (C3S) Climate Data Store (https://cds.climate.copernicus.eu/datasets). The 30-m annual land cover dataset for the Yangtze River Delta (2002–2022) was extracted from the national dataset produced by Yang and Huang (2021), available at https://zenodo.org/records/18180184.
• Code: This paper does not report original code.
• Additional information: Any additional information required to reanalyze the data reported in this paper is available from the lead contact upon request.

## Acknowledgments

This work was supported by the China Meteorological Administration Xiong’an Atmospheric Boundary Layer Key Laboratory (grant no. 2023LABL-B14), the Natural Science Foundation of the Jiangsu Higher Education Institutions of China (grant no. 25KJB170026), Open Research Program of China Meteorological Administration Aerosol-Cloud and Precipitation Key Laboratory (grant no. KDW2401), Ningbo Public Welfare Science and Technology Project (grant nos. 2025S108 and 2025S124), and the Startup Foundation of Nantong University, China (grant no. 135423612053).

## Author contributions

Z.D., writing – original draft, visualization, validation, methodology, investigation, formal analysis, data curation, and conceptualization; S.F., writing – review & editing, formal analysis, resources, and funding acquisition; Y.Z., writing – review & editing, resources, and funding acquisition; T.J., writing – review & editing, formal analysis, and methodology.

## Declaration of interests

The authors declare no competing interests.

## STAR★Methods

### Key resources table

**Table:** 

REAGENT or RESOURCE	SOURCE	IDENTIFIER
**Deposited data**		

Automatic weather station data	China Meteorological Data Network	http://data.cma.cn/
ERA5 Reanalysis dataset	European Center for Medium-Range Weather Forecasts	https://cds.climate.copernicus.eu/datasets

**Software and algorithms**		

ArcGIS 10.8	Environmental Systems Research Institute, Inc.	https://www.esri.com
PyCharm 2025.2.0.1	JetBrains, 2025	https://www.jetbrains.com/zh-cn/pycharm/
Code	Authors	From the lead contact upon request

### Experimental model and study participant details

This study did not involve experimental models or study participants.

### Method details

#### Data

Daily maximum (*T*_max_) and minimum (*T*_min_) temperature observations were obtained from 186 meteorological stations across the Yangtze River Delta region (26°*N*–35°N, 114°E−123°E) for the extended summer period (June–September) spanning 2002–2022. These data were provided by the China Meteorological Administration (http://data.cma.cn/) and underwent homogenization following the methodology described Xu et al. (2013).^48^

To investigate the energy budget characteristics during regional heatwave events, ERA5 reanalysis products from the European Center for Medium-Range Weather Forecasts (ECMWF) were employed, accessed through the Copernicus Climate Change Service (C3S) Climate Data Store (https://cds.climate.copernicus.eu/datasets). The following hourly variables at 0.25° × 0.25° horizontal resolution were analyzed: total cloud cover, 2-m dewpoint temperature (K), surface pressure (Pa), surface downward shortwave radiation flux (W m^−2^), surface downward longwave radiation flux (W m^−2^), surface latent heat flux (W m^−2^), surface sensible heat flux (W m^−2^), soil temperature at level 1 (0–7 cm depth, K), volumetric soil water content at layer 1 (0–7 cm depth, m^3^ m^−3^), boundary layer height (m), and friction velocity (m s^−1^). The 2-m specific humidity was derived from dewpoint temperature and surface pressure using standard thermodynamic relationships. In addition, the 30 m annual land cover data for the Yangtze River Delta from 2002 to 2022 was derived from the China Land Cover Dataset developed by Yang and Huang (2021),^49^ which is openly available at https://zenodo.org/records/18180184.

#### Definition of daytime, nighttime, and compound heatwaves

Heatwave events were identified using daily maximum (*T*_max_) and minimum (*T*_min_) temperatures, following established approaches (Chen & Zhai, 2017; Su & Dong, 2019; Thomas et al., 2020).^25^^,^^50^^,^^51^ As schematically illustrated in Figure S5, a compound heatwave is defined as a period of at least three consecutive days during which both *T*_max_ and *T*_min_ exceed their calendar-day 90th-percentile thresholds. Daytime-only heatwaves occur when *T*_max_ exceeds its 90th-percentile threshold while *T*_min_ remains below its corresponding threshold for three or more consecutive days. Nighttime-only heatwaves are analogously defined by elevated *T*_min_ and sub-threshold *T*_max_ for the same duration. To enhance temporal robustness while preserving seasonality, calendar-day 90th-percentile thresholds were computed for each station using a 15-day moving window (±7 days) over the 2002–2022 reference period, yielding station-specific daily thresholds. Regional heatwaves were identified using spatially averaged time series of daily maximum (*T*_max_) and minimum (*T*_min_) temperatures across all stations within the study area. Based on these regional aggregates, we detected 11 compound events, 7 daytime-only events, and 10 nighttime-only events over the Yangtze River Delta region of China during 2002–2022.

### Quantification and statistical analysis

Hourly ERA5 variables were aggregated into daytime (11:00–16:00 local time) and nighttime (23:00–04:00 local time) averages following established methodologies.^52^^,^^53^^,^^54^ These windows were chosen to represent the quasi-steady states of maximum solar heating and stable nocturnal radiative cooling, respectively, minimizing the influence of rapid transitions around sunrise and sunset.^55^ For every identified heatwave event, both daytime and nighttime means were computed irrespective of the event classification (daytime-only, nighttime-only, or compound). Anomalies for each variable and for both daytime and nighttime means were calculated by removing the climatological seasonal cycle, defined as the multi-year mean over 2002–2022 using a 15-day moving window (±7 days) centered on each calendar day to preserve seasonality while suppressing day-to-day noise. Event composites were then constructed following established practice (e.g., Luo & Lau, 2017)^56^ in two steps: first, daily anomalies were averaged across all days within each event to obtain an event-level anomaly; second, these event-level anomalies were averaged with equal weighting across all events of a given type to yield composite anomalies for compound, daytime-only, and nighttime-only heatwaves. Spatially, composite anomalies of the selected ERA5 variables were obtained by area-averaging for each heatwave category. The statistical significance of these area-mean anomalies was also assessed using a Student’s *t* test at the 95% confidence level to ensure that the regional signals are robust rather than arising from random variability.
