# Supplementary material for: Contrasting boundary-layer energy budgets during daytime, nighttime, and compound heatwaves in eastern China
Source: iScience. 2026 Feb 4;29(3):114892. doi: 10.1016/j.isci.2026.114892 (PMC12933621; doi:10.1016/j.isci.2026.114892)
Supplement: Document S1. Figures S1–S5 [file mmc1.pdf]

iScience, Volume *29*

Supplemental information

Contrasting boundary-layer energy budgets during daytime, nighttime, and compound heatwaves in eastern China

Zexia Duan, Sihui Fan, Yichi Zhang, and Tianbo Ji

iScience, Volume ■ ■

Supplemental information

Contrasting boundary-layer energy budgets during daytime, nighttime, and compound heatwaves in eastern China

Zexia Duan, Sihui Fan, Yichi Zhang, and Tianbo Ji

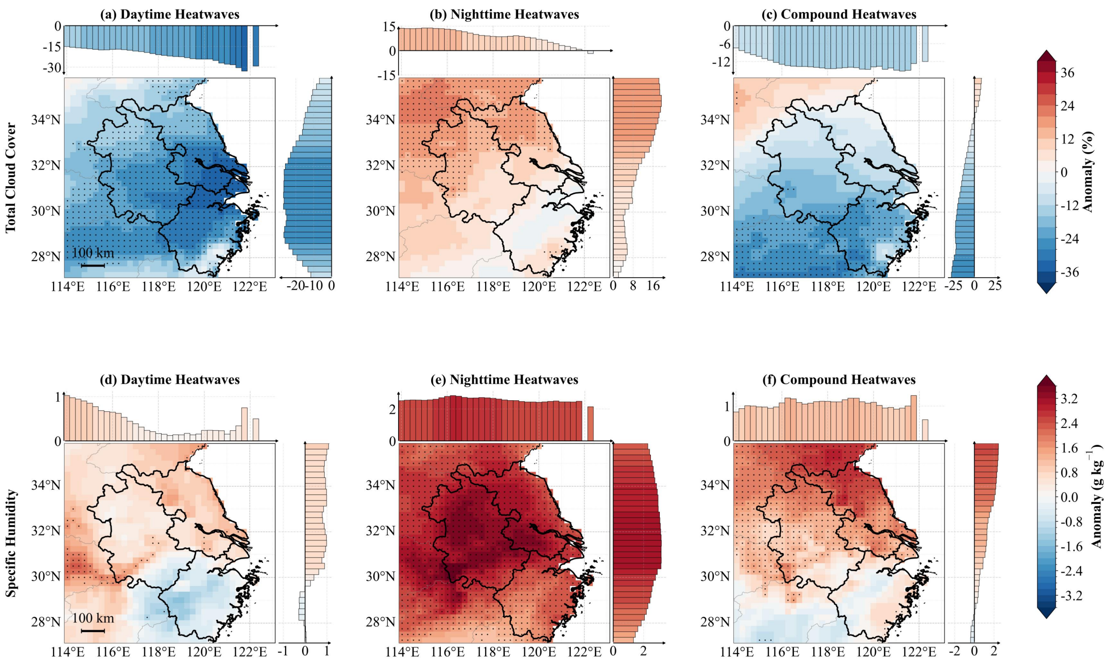

Figure S1. Spatial composite anomalies during nighttime hours (23:00–04:00 local time) for three heatwave types over the Yangtze River Delta (YRD), China.

(a–c) Total cloud cover anomalies.

(d – f) Near-surface specific humidity anomalies. Columns from left to right correspond to daytime-only, nighttime-only, and compound heatwaves. Black stippling on maps marks grid cells where anomalies are statistically significant at the 95% confidence level according to a Student's t-test. Bar plots above and to the right of each map show zonal (longitude-dependent) and meridional (latitude-dependent) variations of the anomalies, respectively. The maps and accompanying bar plots share the same colorbar. Data are represented as the mean anomalies. Scale bars: 100 km.

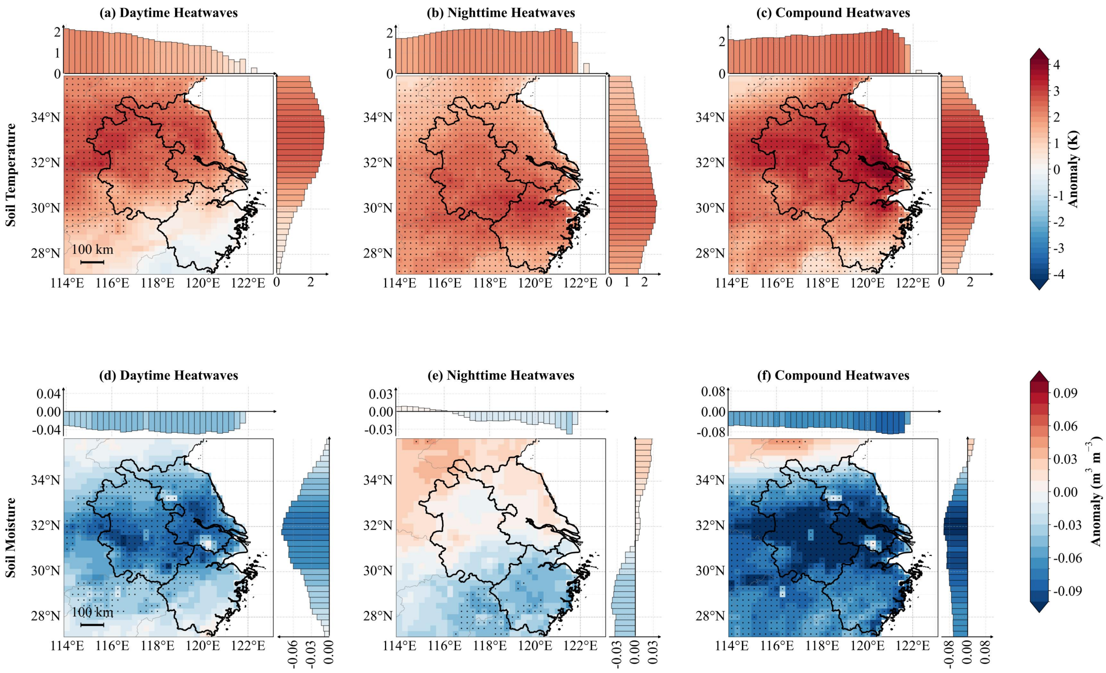

Figure S2. Nighttime (23:00–04:00 local time) soil temperature and soil moisture anomalies.

(a–c) Soil temperature anomalies.

(d–f) Soil moisture anomalies.

In all rows, the columns from left to right correspond to daytime-only, nighttime-only, and compound heatwaves, following the same layout as in Figure S1.

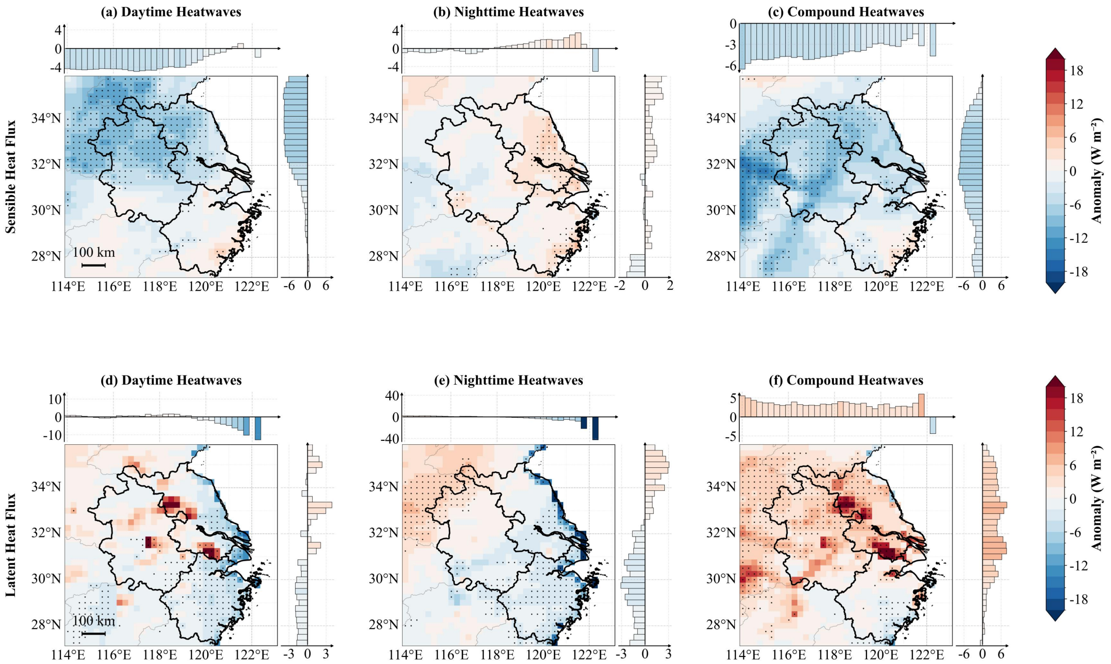

Figure S3. Nighttime (23:00–04:00 local time) sensible heat flux and latent heat flux anomalies.

(a–c) Sensible heat flux anomalies.

(d–f) Latent heat flux anomalies.

In all rows, the columns from left to right correspond to daytime-only, nighttime-only, and compound heatwaves, following the same layout as in Figure S1.

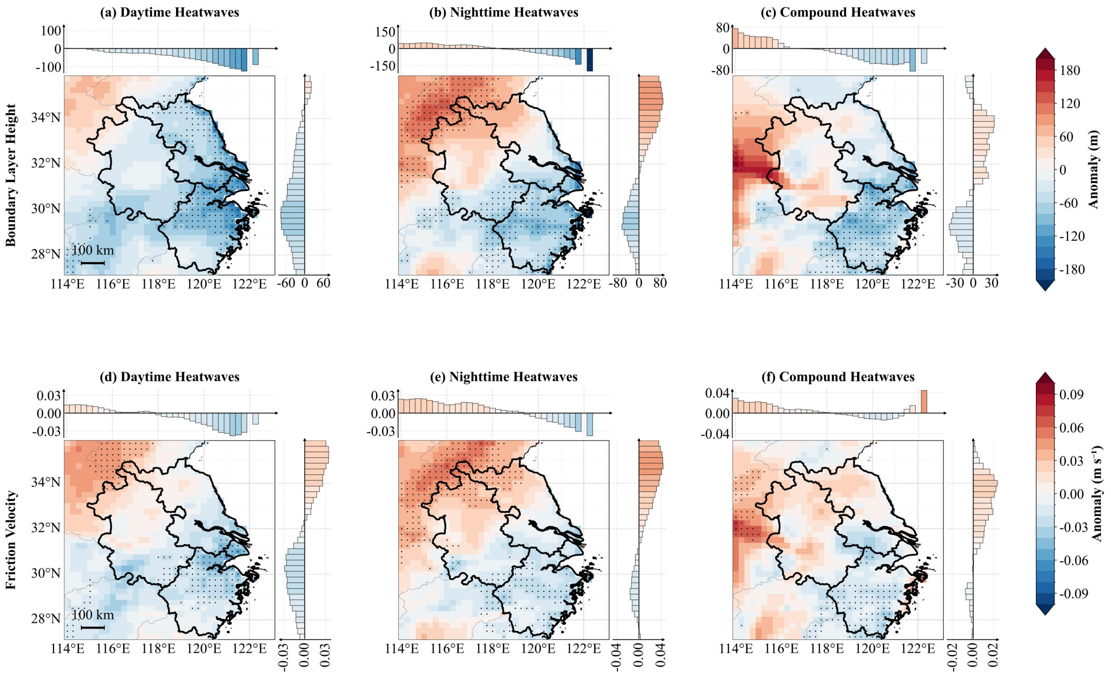

Figure S4. Nighttime (23:00 – 04:00 local time) boundary layer height and friction velocity anomalies.

(a–c) Boundary layer height anomalies.

(d–f) Friction velocity anomalies.

In all rows, the columns from left to right correspond to daytime-only, nighttime-only, and compound heatwaves, following the same layout as in Figure S1.

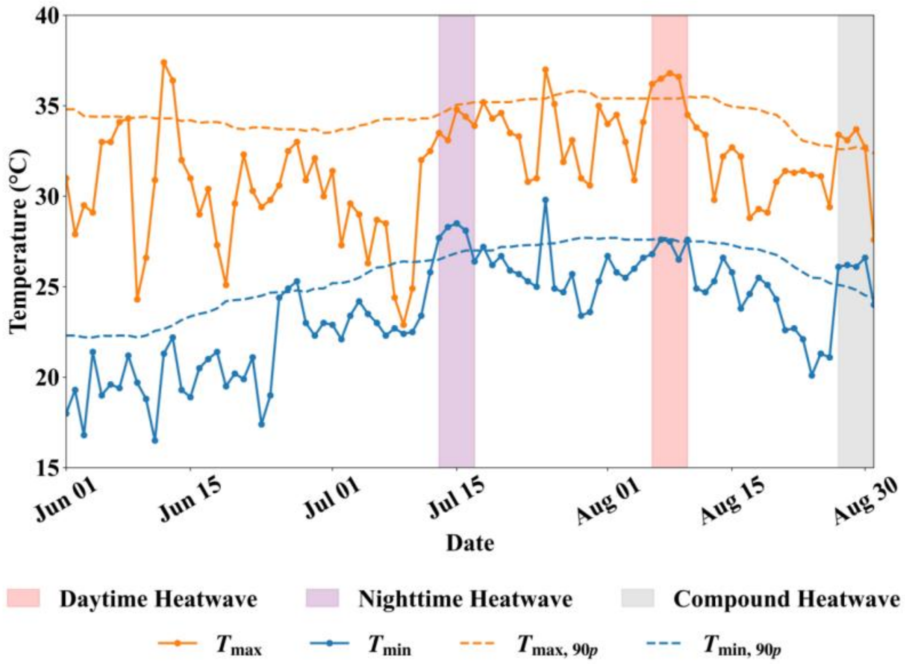

Figure S5. Schematic illustration of daytime-only, nighttime-only, and compound heatwaves.

These events are defined based on whether daily maximum (T_{max}) and minimum (T_{min}) temperatures exceed their corresponding 90th percentile thresholds.
